# Long-term sequelae of SARS-CoV-2 two years following infection: exploring the interplay of biological, psychological, and social factors

**DOI:** 10.1017/S0033291724002721

**Published:** 2024-11

**Authors:** Anouk Verveen, Fajar Agung Nugroho, Ioan Gabriel Bucur, Elke Wynberg, Hugo D.G. van Willigen, Udi Davidovich, Anja Lok, Eric P. Moll van Charante, Godelieve J. de Bree, Menno D. de Jong, Neeltje Kootstra, Tom Claassen, Marien I. de Jonge, Tom Heskes, Maria Prins, Hans Knoop, Pythia T. Nieuwkerk, the RECoVERED Study Group, Ivette Agard, Jane Ayal, Floor Cavdar, Marianne Craanen, Annemarieke Deuring, Annelies van Dijk, Ertan Ersan, Laura del Grande, Joost Hartman, Nelleke Koedoot, Tjalling Leenstra, Romy Lebbink, Dominiqu Loomans, Agata Makowska, Tom du Maine, Ilja de Man, Amy Matser, Lizenka van der Meij, Marleen van Polanen, Maria Oud, Clark Reid, Leeann Storey, Marc van Wijk, Joost van den Aardweg, Joyce van Assem, Marijne van Beek, Thyra Blankert, Maartje Dijkstra, Orlane Figaroa, Leah Frenkel, Marit van Gils, Jelle van Haga, Xiaochuan (Alvin) Han, Agnes Harskamp-Holwerda, Mette Hazenberg, Soemeja Hidad, Nina de Jong, Neeltje Kootstra, Lara Kuijt, Colin Russell, Karlijn van der Straten, Annelou van der Veen, Bas Verkaik, Gerben-Rienk Visser

**Affiliations:** 1 Department of Medical Psychology, Amsterdam UMC, Amsterdam Public Health Research Institute, University of Amsterdam, Amsterdam, the Netherlands; 2 Department of Data Science, Institute for Computing and Information Sciences, Radboud University, Nijmegen, the Netherlands; 3 Department of Informatics, Faculty of Science and Mathematics, Diponegoro University, Semarang, Indonesia; 4 Department of Infectious Diseases, Public Health Service of Amsterdam, Amsterdam, the Netherlands; 5 Department of Infectious Diseases, Amsterdam UMC, University of Amsterdam, Amsterdam Institute for Infection and Immunity, Amsterdam, the Netherlands; 6 Department of Medical Microbiology & Infection Prevention, Amsterdam UMC, University of Amsterdam, Amsterdam Institute for Infection and Immunity, Amsterdam, the Netherlands; 7 Department of Social Psychology, University of Amsterdam, Amsterdam, the Netherlands; 8 Department of Psychiatry, Amsterdam UMC, University of Amsterdam, Amsterdam, The Netherlands; 9 Department of Public & Occupational Health, Amsterdam UMC, Amsterdam Public Health Research Institute, University of Amsterdam, Amsterdam, the Netherlands; 10 Department of General Practice, Amsterdam UMC, Amsterdam Public Health Research Institute, University of Amsterdam, Amsterdam, the Netherlands; 11 Department of Experimental Immunology, Amsterdam UMC, University of Amsterdam, Amsterdam Institute for Infection and Immunity, Amsterdam, the Netherlands; 12 Department of Laboratory Medicine, Laboratory of Medical Immunology, Radboud University Medical Center, Nijmegen, the Netherlands; 13 Center for Infectious Diseases, Radboud University Medical Center, Nijmegen, the Netherlands

**Keywords:** causal discovery, concentration problems, COVID-19, depression, fatigue, HRQL, infection, structural network

## Abstract

**Background:**

Severe fatigue and cognitive complaints are frequently reported after SARS-CoV-2 infection and may be accompanied by depressive symptoms and/or limitations in physical functioning. The long-term sequelae of COVID-19 may be influenced by biomedical, psychological, and social factors, the interplay of which is largely understudied over time. We aimed to investigate how the interplay of these factors contribute to the persistence of symptoms after COVID-19.

**Methods:**

RECoVERED, a prospective cohort study in Amsterdam, the Netherlands, enrolled participants aged⩾16 years after SARS-CoV-2 diagnosis. We used a structural network analysis to assess relationships between biomedical (initial COVID-19 severity, inflammation markers), psychological (illness perceptions, coping, resilience), and social factors (loneliness, negative life events) and persistent symptoms 24 months after initial disease (severe fatigue, difficulty concentrating, depressive symptoms and limitations in physical functioning). Causal discovery, an explorative data-driven approach testing all possible associations and retaining the most likely model, was performed.

**Results:**

Data from 235/303 participants (77.6%) who completed the month 24 study visit were analysed. The structural model revealed associations between the putative factors and outcomes. The outcomes clustered together with severe fatigue as its central point. Loneliness, fear avoidance in response to symptoms, and illness perceptions were directly linked to the outcomes. Biological (inflammatory markers) and clinical (severity of initial illness) variables were connected to the outcomes only via psychological or social variables.

**Conclusions:**

Our findings support a model where biomedical, psychological, and social factors contribute to the development of long-term sequelae of SARS-CoV-2 infection.

## Introduction

Severe fatigue and cognitive complaints, mainly difficulty concentrating and memory problems, are frequently reported after SARS-CoV-2 infection (Ceban et al., 2022; Poole-Wright et al., 2023; Taquet et al., 2022) and may be accompanied by depressive symptoms and/or limitations in physical functioning (Bourmistrova, Solomon, Braude, Strawbridge, & Carter, 2022; Taquet et al., 2022). Known biological risk factors for these persistent symptoms following COVID-19 include female sex, pre-existing somatic and psychological comorbidities, and obesity (Davis, McCorkell, Vogel, & Topol, 2023; Desgranges et al., 2022; Fernández-de-las-Peñas et al., 2022b). The level of initial COVID-19 disease severity, ranging from asymptomatic to critical illness requiring intensive care unit (ICU) care, is also associated with an increased risk of persistent symptoms (Maglietta et al., 2022). Inflammatory cytokine responses are considered to be of clinical relevance, as early inflammatory levels of CRP, IL-1*β*, IL-6, and TNF-*α* have been shown to be associated with post-COVID symptoms (Lai et al., 2023; Schultheiß et al., 2022).

Psychological factors possibly influencing the long-term sequelae of COVID-19 include beliefs related to the illness, which are key determinants of behaviour directed at managing illness or well-being across diseases (Petrie & Weinman, 2006). Reporting persistent symptoms after SARS-CoV-2 infections has been shown to be associated with more serious illness perceptions over time (Hüfner et al., 2023; Wynberg et al., 2023). Additionally, a previous study on individuals with persistent symptoms after COVID-19 showed that illness perceptions explain between 28% and 37% of the variance across health outcomes such as fatigue and mental health problems (Bierbauer, Luscher, & Scholz, 2022). Furthermore, social isolation during COVID-19 lockdown restrictions increased loneliness, which is associated with worse mental health (Killgore, Cloonan, Taylor, & Dailey, 2020). In general, cognitive emotional regulation strategies or coping styles, play an important role in buffering the experience of negative live events and (mental) health complaints (Garnefski, Kraaij, & Spinhoven, 2001). Psychological resilience, the ability to adapt to adversity, can mitigate the negative effect of illness on subjective well-being (Smith et al., 2008). Low levels of resilience have previously been associated with higher severity of persistent complaints after COVID-19 (Bahmer et al., 2022).

Although associations with some of these factors have been described for individuals with persistent symptoms separately, the interplay of biological, psychological, and social factors has not yet been studied in relation to long-term COVID-19 outcomes. In this prospective cohort study, we aimed to determine whether psychological (illness perceptions, cognitive-behavioral responses to symptoms, coping style, resilience), social (loneliness, negative life events), and biomedical factors (disease severity in the acute phase, inflammatory markers) contribute to the persistence of symptoms (fatigue, difficulty concentrating, depression, physical functioning) 24 months after COVID-19 onset and how these factors interact with each other. Insight into which factors play a crucial role in the persistence of these sequelae would enable health professionals and policy makers to develop tailored interventions for key target populations.

## Methods

### Study design and participants

RECoVERED is a cohort study of individuals with SARS-CoV-2 infection in Amsterdam, the Netherlands. Enrolment took place from May 2020 to June 2021 (Nieuwkerk, de Jong, de Bree, Prins, & Visser, 2024; Wynberg et al., 2021). Non-hospitalized participants were identified from notification data of laboratory-confirmed SARS-CoV-2 infection at the Public Health Service of Amsterdam and enrolled within 7 days of diagnosis. Prospectively enrolled hospitalized participants were identified from admissions to the COVID-19 wards of the Amsterdam University Medical Centre and enrolled within 7 days of admission. Up to 30 June 2020, a limited number of hospitalized patients were retrospectively included within 3 months following SARS-CoV-2 diagnosis. None of the participants had been vaccinated for COVID-19 prior to enrolment.

Eligibility criteria included laboratory confirmation of SARS-CoV-2 infection by reverse transcriptase polymerase chain reaction (RT-PCR), age 16–85 years, residence in the municipal region of Amsterdam, and adequate understanding of Dutch or English. Nursing home residents and individuals with mental disorders were excluded. For the present analyses, we included all participants with questionnaire data available on month 24.

RECoVERED was approved by the medical ethical review board of the Amsterdam University Medical Centre (NL73759.018.20). All participants provided written informed consent. The authors asserted that all procedures contributing to this work comply with the ethical standards of the relevant national and institutional committees on human experimentation and with the Helsinki Declaration of 1975, as revised in 2008.

### Study procedures and instruments

Past medical history and socio-demographic data were collected during the first month of follow-up. Physical measurements (i.e. heart rate, respiratory rate [RR], oxygen saturation [SpO_2_]), were measured at days 0 and 7, or retrieved from hospital records for retrospectively enrolled participants.

#### Definitions

Clinical severity groups were defined based on World Health Organization (WHO) COVID-19 severity criteria (World Health Organization, 2021): mild disease as having a RR <20/min and SpO_2_ >94% on room air at both D0 and D7; moderate disease as having a RR 20–30/min and/or SpO_2_ 90–94% or receiving oxygen therapy at D0 or D7; severe disease as having a RR>30/min and/or SpO_2_<90% or receiving oxygen therapy at D0 or D7; critical disease as ICU admission due to COVID-19 at any point. Comorbidities were those associated with more severe COVID-19 based on World Health Organization Clinical Management Guidelines (World Health Organization, 2021) and include cardiovascular disease (including hypertension), chronic pulmonary disease (excluding asthma), renal disease, liver disease, cancer, immunosuppression, and psychiatric illness. Migration background was categorized as Dutch or non-Dutch based on the country of birth of the participant and their parents (Centraal Bureau voor de Statistiek; Stronks, Kulu-Glasgow, & Agyemang, 2009); those of non-Dutch background were further classified as originating from a high-income (HIC) or low-/middle-income country (LMIC) (OECD, 2021). The highest attained educational level was categorized as: none; primary/secondary school; vocational training; university-level.

#### Measurements

Participants completed online questionnaires consisting of different combinations of validated measures at months 1, 3, 6, 9, 12, 18, and 24. All outcomes of the current study (outlined below) were assessed at months 12 and 24.

*Outcome measures*. Severe fatigue and difficulty concentrating were assessed using the Checklist Individual Strength (CIS) (Vercoulen et al., 1994). A total CIS fatigue subscale score (8 items, total range 8–56) of ⩾35 defines the presence of severe fatigue (Vercoulen et al., 1994; Worm-Smeitink et al., 2017). On the concentration subscale (5 items, range 5–35) a threshold of ⩾18 defines the presence of notable concentration problems (Verveen et al., 2023; Worm-Smeitink et al., 2017).

The Patient Health Questionnaire-9 (PHQ-9) scores the nine DSM criteria of depression on a scale from 0 (never present) to 3 (present nearly every day), where higher scores indicate higher levels of depressive symptoms (Kroenke, Spitzer, Williams, & Lowe, 2010; Spitzer, Kroenke, Williams, & Primary, 1999). A cutoff point of 10 indicates the presence of clinically relevant symptoms of depression.

The Medical Outcomes Study Short Form 36-item health survey (SF-36) comprises 36 items that address 8 dimensions reflecting the respondent's health-related quality of life (HRQL) from 0 to 100, with higher scores indicating better HRQL (Ware, 1993). In the current analyses, the dimension describing the ability to perform usual and vigorous activities (physical functioning) is used with scores ranging from 0 (maximal limitations) to 100 (no limitations)

*Putative factors*. The Brief Illness Perceptions Questionnaire (B-IPQ) consists of 8 items scored on a 11-point scale with different response options per item (Broadbent, Petrie, Main, & Weinman, 2006). A total score (range 0–80) can be computed where a higher score represents a perception of the illness of being more serious.

The six items of the Brief Resilience Scale (BRS) are scored on a scale from 1 = strongly disagree to 5 = strongly agree and combined into a mean summary score. Higher scores are associated with better resilience (Smith et al., 2008).

Three subscales of Cognitive and Behavioral Responses to Symptoms questionnaire (CBRSQ) (Picariello, Chilcot, Chalder, Herdman, & Moss-Morris, 2023) were used: fear avoidance (6 items, range 0–24, perceived danger of undertaking activities), damage beliefs (5 items, range 0–20, beliefs that symptoms indicate physical damage), and all-or-nothing behavior (5 items, range 0–20, periods of high activity followed by periods of inactivity). Higher scores indicate more fear avoidance/all-or-nothing behavior or stronger damage beliefs.

To define coping styles, a 12-item version of the Cognitive Emotion Regulation Questionnaire (CERQ) is used, consisting of 10 items of the short CERQ (Garnefski & Kraaij, 2006) and 2 items from the Positive Appraisal Style Scale (Veer et al., 2021). The following five subscales are calculated: acceptance, positive refocusing, refocus on planning, positive reappraisal, and putting into perspective.

The shortened De Jong Gierveld loneliness scale (DJGLS) consists of six items where a total score (range 0–6) of 2–4 indicates some loneliness and ⩾5 loneliness (van Beuningen, Coumans, & Moonen, 2018).

Occurrence of nine negative life events (NLE) in the 12 months preceding the SARS-CoV-2 infection was collected: adverse change in health status; adverse change in health status of significant other or close relative; passing of a child, sibling, parent or partner; passing of a close relative or good friend; adverse change in a romantic relationship; relationship problems; conflict with a friend, neighbor or relative; loss of employment; severe financial difficulty (Acarturk et al., 2009).

The putative factors were assessed at the following follow-up measurements: negative life events, month 1; DJGLS and CBRSQ, month 3; BRS and CERQ, month 6; B-IPQ, month 12.

#### Inflammation markers

Serum samples for cytokine analysis were collected at day 0 and subsequently at months 1, 3, and 6. C-reactive protein (CRP), soluble CD14, soluble CD163, tumor necrosis factor (TNF)-*α*, interferon-*γ*-inducible protein 10 (IP-10)/CXCL10, monocyte chemoattractant protein (MCP)1, interleukin (IL)-1*β*, IL-2, IL-6, IL-10, IL-13, and IL-17A, concentrations were analyzed in serum with human magnetic Luminex screening assays (LXSAHM-02 and LXSAHM-10; R&D Systems). Assays were performed according to the manufacturer's instructions.

### Network and statistical analysis

Socio-demographic and clinical characteristics of participants were compared between clinical severity groups using Kruskal–Wallis and Mann–Whitney *U* tests. Two-sided *p* values <0.05 were considered statistically significant.

**Figure 1.**
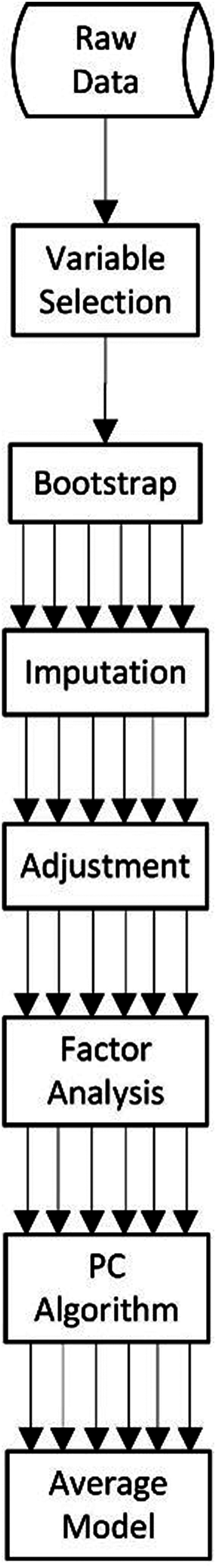
Flowchart methods. Single arrows indicate one process, whereas multiple arrows represent the analyses performed on 500 bootstrap samples of the original dataset. PC, Peter and Clark.

The data preparation and analysis are visualized in a flowchart (Fig. 1). We started the analysis by conducting bootstrap sampling to create 500 resampled datasets, to account for model fitting uncertainty. In each individual bootstrap sample, missing data was imputed using MICE imputation (van Buuren & Groothuis-Oudshoorn, 2011). We then performed factor analysis to combine related subscales in the CERQ questionnaire, with the goal of simplifying the data and capturing the overall relationships between psychological latent concepts and the outcomes of interest. We performed maximum-likelihood factor analysis using the *factanal* function from the *stats* R package (version 4.3.1) (R Core Team, 2013). After these preprocessing steps, within each bootstrap dataset, we conducted linear regression on each variable selected for analysis to adjust for the following sociodemographic baseline variables: age (continuous), BMI (continuous), smoking (non-smoker; smoker; ex-smoker), migration background, education level, and comorbidities (see definitions for categories).

After baseline covariate adjustment, 500 structural models were produced using the Peter and Clark (PC) causal discovery algorithm (Spirtes, Glymour, & Scheines, 2000) from the *pcalg* R package (Kalisch, Mächler, Colombo, Maathuis, & Bühlmann, 2012). The structural model consists of a network of variables and links (direct associations between the variables that cannot be fully mediated by any other variable(s) in the model). We averaged the links obtained in the 500 models with the bootstrap stability threshold of 0.5 to produce the final output model. The 0.5 threshold corresponds to a majority decision, ensuring that any links connecting two variables exist in at least half of the fitted models. The stability of a link between variables in the network is represented by the thickness of the link. A link present in 50–80% of the 500 models has low stability, between 81–97% moderate stability, and >97% high stability. A correlation matrix was computed to indicate the positive or negative sign of all the association links in the models. Note that a significant correlation between two variables does not automatically imply a stable link, as the variables can be connected through multiple (direct or indirect) pathways. The network analysis also shows us which of these pathways are more stable. After the structural model was completed, we used the data-driven PC algorithm to determine if stable links between variables were causal, again with a threshold of 0.5.

The main analysis includes continuous outcomes measured at month 24 and inflammatory markers at month 3. The same procedure was also run under two different variable selection scenarios. Specifically, in a first sensitivity analysis, we considered outcome variables measured at month 12 of follow-up instead of at month 24, while in a second sensitivity analysis, we considered inflammatory marker samples from month 6 instead of from month 3. Finally, this procedure was run for the outcome variables severe fatigue and concentration separately, as these are frequently reported after SARS-CoV-2 infection (Ceban et al., 2022).

## Results

### Study population

Of 303 participants enrolled in RECoVERED, 235 participants completed the month 24 study visit and were included in this study (77.6%). Participants who were lost to follow up and excluded in the analyses (*n* = 68, 22.4%) did not differ significantly in age, sex, BMI, educational level, smoking status, or number of comorbidities from included participants (Supplementary Table 1). Participants who were lost to follow up were more likely to have been hospitalized due to SARS-CoV-2 infection than included participants (68% *v.* 43%, *p* < 0.001).

Table 1 shows demographic and clinical characteristics of the participants included in this study. Total scores and cutoff scores for the outcomes (fatigue, concentration, depressive symptoms, and physical functioning) and putative factors (B-IPQ, BRS, CBRSQ, CERQ, DJGLS, NLE, and inflammation markers) are presented in Table 2. Subscales from the CERQ were combined into one variable using factor analysis.

**Table 1.** Socio-demographic and clinical characteristics of RECoVERED study participants

	Levels	Total	Initial mild COVID-19	Initial moderate COVID-19	Initial severe/critical COVID-19	*p* value
Total *N* (%)		235	70 (29.8)	107 (45.5)	58 (24.7)	
Sex	Male	137 (58.3)	36 (51.4)	64 (59.8)	37 (63.8)	0.336
	Female	98 (41.7)	34 (48.6)	43 (40.2)	21 (36.2)	
Age, years	Median (IQR)	52.0 (38.0–62.0)	46.0 (31.0–55.0)	51.0 (35.0–61.5)	60.0 (52.2–65.0)	<0.001
BMI, kg/m^2^	Median (IQR)	25.8 (23.2–29.3)	24.6 (22.9–27.6)	25.6 (23.5–29.1)	27.2 (24.9–31.0)	0.011
BMI category	Normal weight	101 (43.0)	40 (57.1)	45 (42.1)	16 (27.6)	0.023
	Overweight	82 (34.9)	20 (28.6)	38 (35.5)	24 (41.4)	
	Obese	51 (21.7)	10 (14.3)	24 (22.4)	17 (29.3)	
	(Missing)	1 (0.4)	0 (0.0)	0 (0.0)	1 (1.7)	
Migration background^a^	Dutch	156 (66.4)	53 (75.7)	65 (60.7)	38 (65.5)	0.083
	Non-Dutch, OECD HIC	28 (11.9)	8 (11.4)	16 (15.0)	4 (6.9)	
	Non-Dutch, OECD LMIC	50 (21.3)	8 (11.4)	26 (24.3)	16 (27.6)	
	(Missing)	1 (0.4)	1 (1.4)	0 (0.0)	0 (0.0)	
Education level	No formal education	33 (14.0)	4 (5.7)	22 (20.6)	7 (12.1)	<0.001
	Primary education	54 (23.0)	7 (10.0)	23 (21.5)	24 (41.4)	
	Secondary education	147 (62.6)	58 (82.9)	62 (57.9)	27 (46.6)	
	(Missing)	1 (0.4)	1 (1.4)	0 (0.0)	0 (0.0)	
Number of COVID-19 high-risk comorbidities^b^	0	129 (54.9)	48 (68.6)	64 (59.8)	17 (29.3)	<0.001
	1	58 (24.7)	16 (22.9)	22 (20.6)	20 (34.5)	
	2	32 (13.6)	5 (7.1)	15 (14.0)	12 (20.7)	
	3	16 (6.8)	1 (1.4)	6 (5.6)	9 (15.5)	
Smoking status	Non-smoker	143 (60.9)	41 (58.6)	61 (57.0)	41 (70.7)	0.249
	Smoker	16 (6.8)	7 (10.0)	8 (7.5)	1 (1.7)	
	Ex-smoker	75 (31.9)	21 (30.0)	38 (35.5)	16 (27.6)	
	(Missing)	1 (0.4)	1 (1.4)	0 (0.0)	0 (0.0)	

BMI, body mass index; COVID-19, coronavirus disease 2019; HIC, high-income country; LMIC, low- and middle-income country; OECD, Organisation for Economic Co-operation, and Development.

COVID-19 clinical severity groups defined as: mild as having a respiratory rate (RR) <20/min and oxygen saturation (SpO_2_)on room air >94% at both days 0 and 7; moderate disease as having a RR 20––30/min, SpO_2_ 90–94% and/or receiving oxygen therapy at days 0 or 7; severe disease as having a RR >30/min or SpO_2_ < 90% at days 0 or 7; critical disease as requiring intensive care unit admission.

a Migration background was based on country of birth of participant and that of their parents and included first and second-generation migrants.

b COVID-related comorbidities are based on World Health Organization Clinical Management Guidelines (World Health Organization, 2021) and include: cardiovascular disease (including hypertension), chronic pulmonary disease (excluding asthma), renal disease, liver disease, cancer, immunosuppression (excluding HIV, including previous organ transplantation), previous psychiatric illness and dementia.

**Table 2.** Outcomes and putative factors

	Total	Initial mild COVID-19	Initial moderate COVID-19	Initial severe/critical COVID-19	*p* value
Total *N* (%)	235	70 (29.8)	107 (45.5)	58 (24.7)	
Outcomes					
Fatigue (CIS, M24)	28.0 (16.0–38.8)	18.0 (9.0–31.0)	30.0 (19.5–39.5)	33.0 (20.2–43.8)	0.002
Severe fatigue (CIS ⩾35, M24)	47 (20.0)	9 (12.9)	23 (21.5)	15 (25.9)	0.035
*Missing*	85 (36.2)	21 (30.0)	40 (37.4)	24 (41.4)	
Concentration problems (CIS, M24)	14.0 (9.0–21.0)	11.0 (8.0–17.0)	14.0 (9.5–22.0)	16.5 (10.0–23.0)	0.116
Difficulty concentrating (CIS ⩾18, M24)	10 (20.4)	26 (38.8)	15 (44.1)	51 (34.0)	0.043
*Missing*	85 (36.2)	21 (30.0)	40 (37.4)	24 (41.4)	
Depressive symptoms (PHQ-9, M24)	3.0 (1.0–7.0)	2.0 (0.2–6.0)	4.0 (2.0–7.2)	3.0 (2.0-9.8)	0.042
Clinically relevant symptoms of depression (HADS ⩾10, M24)	23 (9.8)	6 (8.6)	8 (7.5)	9 (15.5)	0.077
*Missing*	75 (31.9)	16 (22.9)	35 (32.7)	24 (41.4)	
Physical functioning (SF-36, M24)	95.0 (67.5–100.0)	100.0 (90.0–100.0)	92.5 (60.0–100.0)	82.5 (55.0–95.0)	<0.001
Putative factors					
At least one NLE in the last 12 months (M1)	96 (40.9%)	28 (40.0%)	49 (45.8%)	19 (32.8%)	0.117
Loneliness (DJGLS, M3)	1.0 (0.0–3.0)	1.0 (0.0–2.0)	1.0 (0.0–3.0)	1.0 (0.0–3.0)	0.695
Loneliness (DJGLS ⩾5, M3)	15 (6.4)	4 (5.7)	5 (4.7)	6 (10.3)	0.326
*Missing*	20 (8.5)	6 (8.6)	4 (3.7)	10 (17.2)	
Resilience (BRS, M6)	3.7 (3.0–4.0)	3.7 (3.2–4.2)	3.7 (3.0–4.0)	3.3 (3.0–4.0)	0.189
Illness perceptions (B-IPQ, M12)	27.0 (14.2–40.8)	20.0 (10.8–29.2)	28.5 (17.0–39.8)	41.5 (22.8–47.8)	<0.001
Fear avoidance (CBRSQ, M3)	9.0 (5.5–12.0)	7.0 (4.0–10.0)	9.0 (6.5–13.0)	10.0 (6.8–13.0)	<0.001
Damage avoidance (CBRSQ, M3)	8.0 (6.0–11.0)	7.0 (5.0–9.0)	9.0 (6.0–11.0)	9.0 (6.8–12.0)	<0.001
All-or-nothing behaviour (CBRSQ, M3)	7.0 (5.0–10.0)	7.0 (5.0–9.2)	7.0 (5.0–10.0)	6.0 (4.0–10.2)	0.623
Acceptance (CERQ, M6)	6.0 (5.0–8.2)	8.0 (5.0–9.0)	7.0 (5.0–8.0)	6.0 (4.0–7.0)	0.029
Positive refocusing (CERQ, M6)	6.0 (4.8–8.0)	6.0 (4.0–8.0)	7.0 (5.0–8.0)	5.0 (4.0–7.0)	0.036
Refocus on planning (CERQ, M6)	7.0 (5.0–8.0)	7.0 (5.0–9.0)	8.0 (6.0–8.0)	6.0 (5.0–7.0)	0.002
Positive reappraisal (CERQ, M6)	6.0 (4.0–8.0)	7.0 (5.0–9.0)	7.0 (5.0–8.0)	4.5 (3.0–6.2)	<0.001
Putting into perspective (CERQ, M6)	7.0 (5.0–8.2)	7.0 (5.0–9.0)	7.0 (5.0–8.0)	6.0 (4.0–7.0)	0.023
Inflammation markers (log pg/ml)					
CRP (M3)	6.0 (5.6–6.5)	5.7 (5.3–6.1)	6.0 (5.7–6.7)	6.2 (5.9–6.6)	0.001
sCD14 (M3)	6.1 (6.0–6.3)	6.1 (6.0–6.3)	6.1 (6.0–6.2)	6.2 (6.0–6.4)	0.057
MCP-1 (M3)	2.7 (2.5–2.8)	2.6 (2.5–2.7)	2.7 (2.5–2.8)	2.7 (2.6–2.8)	0.021
IP-10 (M3)	1.2 (1.1–1.4)	1.1 (1.0–1.3)	1.3 (1.1–1.4)	1.4 (1.2–1.6)	<0.001
IL-2 (M3)	0.3 (0.3–0.3)	0.3 (0.3–0.3)	0.3 (0.3–0.3)	0.3 (0.3–0.3)	<0.001
IL-10 (M3)	−1.0 (−1.0 to −0.8)	−1.0 (−1.0 to −0.7)	−1.0 (−1.0 to −0.7)	−1.0 (−1.0 to −1.0)	0.575
IL-17A (M3)	0.0 (0.0–0.7)	0.0 (0.0–0.4)	0.0 (0.0–0.6)	0.4 (0.0–0.7)	0.051
sCD163 (M3)	5.8 (5.6–5.9)	5.7 (5.5–5.8)	5.8 (5.6–5.9)	5.9 (5.7–6.0)	<0.001
IL-1β (M3)	0.1 (0.1–0.9)	0.1 (0.1–0.9)	0.2 (0.1–1.1)	0.1 (0.1–0.2)	0.206
IL-6 (M3)	0.4 (−0.0 to −1.4)	0.2 (−0.3 to −1.1)	0.5 (0.2–1.6)	0.5 (−0.0 to −1.0)	0.115
IL-13 (M3)	1.5 (1.5–1.5)	1.5 (1.5–1.5)	1.5 (1.5–1.5)	1.5 (1.5–1.5)	0.072
TNF-*α* (M3)	0.8 (0.5–1.3)	0.6 (0.4–1.0)	0.9 (0.6–1.3)	0.9 (0.7–1.3)	0.006

B-IPQ, Brief Illness Perceptions Questionnaire; BRS, Brief Resilience Scale; CBRSQ, Cognitive and Behavioral Responses to Symptoms Questionnaire; CERQ, Cognitive Emotion Regulation Questionnaire; CIS, Checklist Individual Strength; CRP, C-reactive protein; DJGLS, De Jong Gierveld loneliness scale; IL, Interleukin; IP-10, Interferon-*γ*-inducible protein; MCP-1, monocyte chemoattractant protein; NLE, negative life events; PHQ-9, Patient Health Questionnaire-9; SF-36, Medical Outcomes Study Short Form 36-item health survey; TNF, tumor necrosis factor.

Continuous variables presented as median (IQR) and compared using the Kruskal–Wallis test; categorical and binary variables presented as *n*(%) and compared using the Mann–Whitney *U* test.

### Structural model

**Figure 2.**
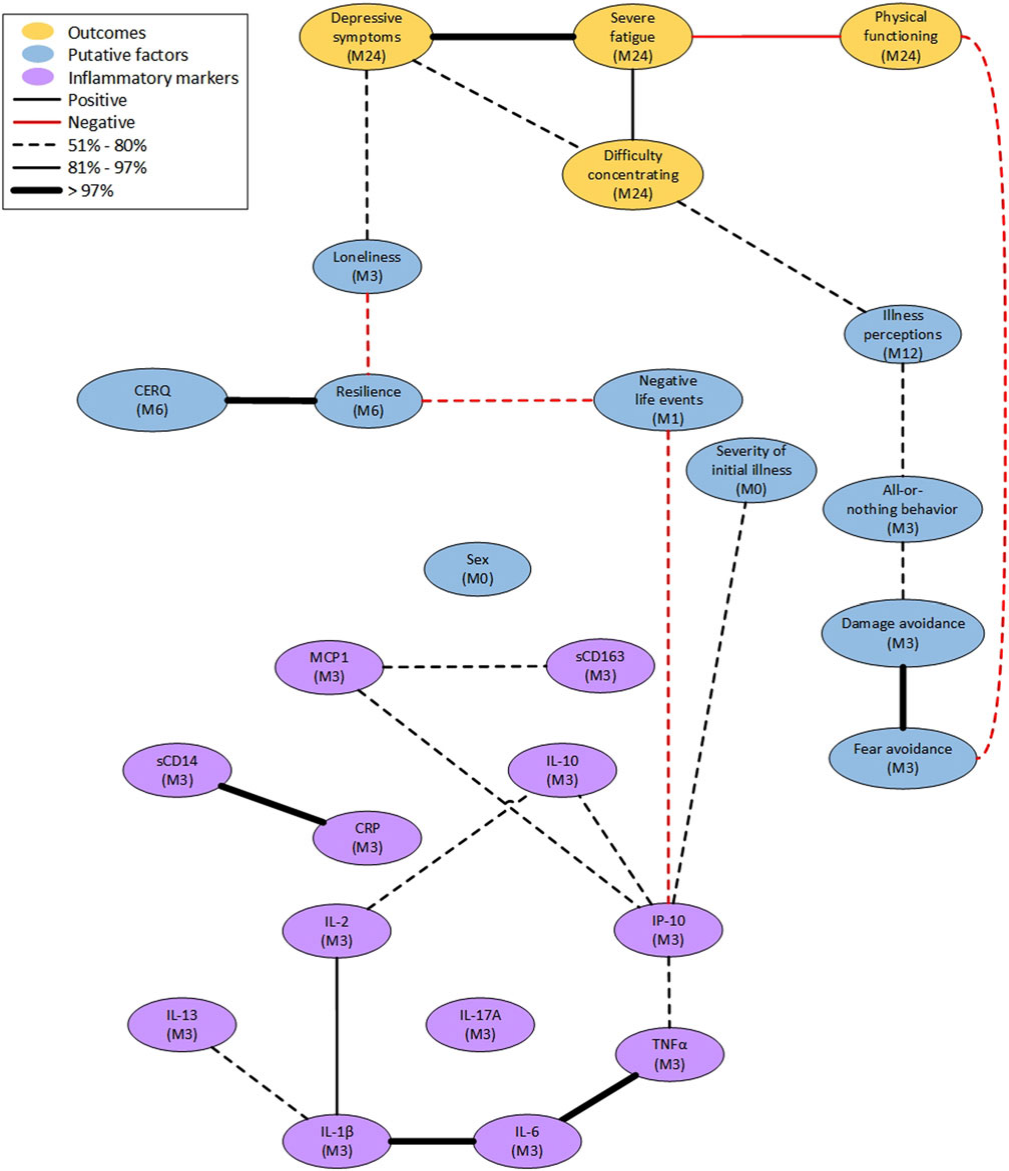
Structural network model. Each line stands for a stable interaction between the two variables it connects, which is not mediated by any other variable in the model. The thickness of a line shows the stability of the interaction: a dashed line has low stability (51–80%), a solid line is moderately stable (81–97%), and a bold line very stable (>97%). Red lines refer to a negative correlation between the two connected variables and black lines to a positive correlation. M# gives the month of measurements. CRP, C-reactive protein; IL, Interleukin; IP, Interferon-*γ*-inducible protein; MCP, Monocyte chemoattractant protein.

The four outcome variables and 26 variables associated to putative factors were included in the main structural network analysis. We observed 25 stable links (non-directional associations) between variables in this main analysis, whose final output model is shown in Fig. 2. The link stability values and the data correlation matrix are presented in the supplementary materials.

The model confirms that variables tend to be grouped based on their origin, i.e., the instrument to which they belong. The outcomes clustered together with severe fatigue as its central point. There are three putative factors linked directly to the outcomes. A higher score on the PHQ-9, indicating more depressive symptoms, is associated with more loneliness. A more serious perception of the illness is positively associated with the other outcomes via concentration problems. Finally, a higher level of fear avoidance is associated with reduced physical functioning.

The inflammatory markers (CRP, sCD14, MCP1, IP-10, IL-2, IL-10, IL-17A, sCD163, IL-1β, IL-6, IL-13, and TNF-α) at month 3 also clustered together. We found two paths within this cluster: the first one is from CRP to sCD14 and the second one is from IL-1β to IL-6 then to TNF-α. The inflammatory markers linked to the outcomes through IP-10. Higher concentration of IP-10 was associated with a lower number of negative life events in the year preceding the onset of COVID-19 and more severe initial COVID-19. Severity of the initial illness does not have links to any other variables. Sex does not have any stable links with other variables in the model. This agrees with the correlation matrix, as these variables are not strongly correlated to any of the outcomes or putative factors.

Regarding the putative factors in the model, the combined CERQ variable, representing coping styles, is directly connected to resilience, and indirectly to loneliness and depressive symptoms. More resilience is associated with lower loneliness scores. Of the CBRSQ variables, representing cognitive and behavioral responses to symptoms, all-or-nothing behavior and fear avoidance are associated with the outcomes, through illness perceptions and physical functioning, respectively. More all-or-nothing behavior is associated with a more serious perception of the illness, while more fear avoidance is associated with reduced physical functioning.

#### Sensitivity analyses

As a sensitivity analysis, the same procedure was performed with outcomes measured at month 12. We found a similar structure as in the main analysis, presented in Supplementary Figure 2. The main difference from the model using month 24 outcomes is that illness perceptions are still connected to the outcomes but no longer to any of the other variables in the model. At month 12, illness perceptions are linked to both severe fatigue and physical functioning instead of concentration problems. Fear avoidance remains directly linked to physical functioning, although the association between all-or-nothing behavior and illness perceptions falls below the threshold. All other links remain present but differ in stability.

In a second sensitivity analysis, we looked at differences between the structural models using inflammatory marker samples from month 6 of follow-up instead of month 3. This model is presented in Supplementary Figure 3. Differences are mostly observed within the cluster of inflammatory markers. Links that were previously connecting IP-10 to severity and MCP1, IL-1β to IL-2, IL-13 to IL-1β and to IL-2 are no longer present. However, a link from MCP1 to sex appears when using month 6 samples for inflammatory markers. Other than that, we now find stable links between IL-13 and IL-17A, between sCD14 and MCP1, and between IP-10 and CRP. The stable links between IL-1β, IL-6, and TNF-α remain.

Last, we looked at differences between the main structural model and models with only one of the outcome measures, namely severe fatigue or concentration. The structure of the models using fatigue (Supplementary Figure 4) and concentration was substantially similar but differed slightly from the main model. The links from loneliness and illness perceptions to the outcomes remained but shifted to the outcome variable selected. In both models, the link with illness perceptions is very stable, while this link had low stability in the main model. Loneliness remains connected to the outcomes with low stability. Additionally, illness perceptions are linked to severity of initial illness in this model. Fear avoidance is not directly linked to severe fatigue, whereas in the main model this variable was linked to the outcomes via physical functioning.

#### Causality

We did not find any causal links with a stability score above 0.5. This suggest that there are no stable causal patterns that can be ascertained from our data sample alone.

## Discussion

In this study we present a structural model of biomedical, psychological, and social factors that contribute to the persistence of fatigue, difficulty concentrating, depressive symptoms and limitations in physical functioning at 24 months after COVID-19 onset. To the best of our knowledge, we are the first to use this approach to gain insight into the factors associated with these outcomes. Similar approaches have been used with outcomes such as chronic back pain (Huie et al., 2022) or alcohol-related cognitive deficits (Fidder et al., 2023).

As expected, the outcomes severe fatigue, difficulty concentrating, depressive symptoms and limitations in physical functioning, clustered together in the structural network, i.e., were strongly related with each other. This co-occurrence of persistent symptoms after COVID-19 has been described previously (Taquet et al., 2021). The results suggest shared underlying mechanisms as in both the main model, as well as the models with specific outcomes, similar links have been found. These underlying factors may be infection-specific, as described in a study on persistent symptoms after Lyme borreliosis (Vrijmoeth et al., 2023). One infection-specific mechanism that we hypothesized to be relevant to long-term outcomes was the inflammatory response (Lai et al., 2023; Schultheiß et al., 2022). However, inflammatory markers at month 3 were not directly associated to the outcomes in our model. In general, we found few links between clinical and biological variables related to COVID-19 that substantially contributed to the network. The only relationships between these variables with the outcomes were via psychological or social variables. For example, the cluster of inflammatory markers at month 3 was associated with the outcomes via the experience of a negative life event in the year prior to infection. Data on other biomedical mechanisms that could possibly be related to post-COVID complaints (Davis et al., 2023) was not collected in this study as they were unknown when the study was initiated, i.e. in May 2020. At that time, the main hypothesis was that the inflammatory response was important.

Others have previously conducted a network analysis of post-COVID pain, resulting in a model where pain was significantly connected to cognitive and psychological variables, including depressive symptoms, and sex (Fernández-de-las-Peñas et al., 2022a). Unexpectedly, we did not find sex to be associated with any of the variables in the model. Within the literature, female sex has consistently been found to be a risk factor for persistent complaints (Fernández-de-las-Peñas et al., 2022b; Rahmati et al., 2023; Tsampasian et al., 2023) and decreased mental health after SARS-CoV-2 infection (Rudenstine et al., 2022). Within our prospective cohort, being female did not consistently emerge as a risk factor for persistent complaints (Verveen et al., 2022; Wynberg et al., 2021). We hypothesize this difference is related to how participants are recruited into studies: prospective or self-referring with persistent symptoms. Women with persistent symptoms may be more inclined to sign-up for studies directed at persistent symptoms than men, whereas our cohort enrolled participants prospectively from illness onset onwards and was therefore able to mitigate this selection bias potentially present in other studies.

Our findings are in line with previous findings from other post-infectious syndromes. Predictors of persistent symptoms after Lyme Borreliosis include impaired physical and social functioning, higher depression and anxiety scores, and more serious illness perceptions at baseline (Vrijmoeth et al., 2023). In chronic Q fever and Q fever fatigue syndrome, illness perceptions, physical and cognitive functioning partially mediated the impact of infection on psychosocial functioning and quality of life years after infection, where fatigue was the main mediator (Reukers et al., 2019). Also following glandular fever, negative illness beliefs were associated with chronic fatigue (Moss-Morris, Spence, & Hou, 2011). It has previously been reported that reporting a greater number of persistent symptoms after COVID-19 was congruent with more serious illness perceptions (Wynberg et al., 2023). In our analysis, all-or-nothing behavior linked to the outcomes through illness perceptions. The link from illness perceptions to the outcomes was consistent across models and was very stable in the sensitivity models with just one outcome. This is likely because in the main model links from illness perceptions to the outcomes in the bootstrap datasets are found randomly spread across the four highly correlated outcomes, lowering the stability of the link. This random spread also became apparent when looking at the link between resilience and the different subscales of the CERQ. After summarizing the CERQ into a single combined factor, the stable link with resilience became visible.

Coping styles and resilience were not directly linked to persistence of symptoms, only through loneliness. Previous studies have described that coping styles that focus on eliminating the negative emotional response to the problem are associated with higher levels of loneliness (Bota et al., 2024; Deckx, van den Akker, Buntinx, & van Driel, 2018). Loneliness has previously been described to contribute to depressive symptoms (Erzen & Cikrikci, 2018), especially during the COVID-19 pandemic (Pai & Vella, 2021). We found associations between loneliness, depression and fatigue. Such relationships were also previously found in the general population due to the COVID-19 pandemic and the consequent lockdowns (Kalfas et al., 2024; Ori, Wieling, Lifelines Corona Research, & van Loo, 2023). Our results therefore not necessarily reflect an effect of having experienced SARS-CoV-2 infection but may also reflect impacts of the pandemic. Social support may have been limited during the pandemic, while this is particularly necessary during dire times to facilitate hope, reduce loneliness (Bareket-Bojmel, Shahar, Abu-Kaf, & Margalit, 2021) and thereby mitigate long-term sequelae such as the outcomes investigated in the current study. An association between loneliness and persistent complaints following COVID-19 has also been found in adolescents (12–25 years) (Selvakumar et al., 2023). Fear avoidance has previously been linked to health outcomes, specifically severe fatigue not following COVID-19, and can be modifiable to relieve symptoms (de Gier et al., 2023).

An unexpected finding was that reporting negative life events in the 12 months prior to infection were related with lower levels of IP-10. Previous studies have shown that the experience of negative life events may result in increased levels of pro-inflammatory markers, mainly IL-6 and CRP, via psychological distress (Baumeister, Akhtar, Ciufolini, Pariante, & Mondelli, 2016; Flouri, Francesconi, Papachristou, Midouhas, & Lewis, 2019). In the present study the relationship was in the opposite direction, as indicated both by the fitted models and the data correlation matrix.

In all versions of the model, links between IL-1β, IL-6, and TNF-α are found with strong stability. Previous research has described that persistent symptoms following COVID-19 are associated with high levels of these three inflammatory markers (Schultheiß et al., 2022). This consistent finding adds to the validity of our structural model.

We were unable to determine causal links between putative factors and outcomes, and we did not find many very stable links between the variables included in the structural model. Variables in the model were not as correlated as expected based on existing literature. This suggests that more research is needed to identify other key variables that could better explain why some people develop these persistent symptoms. Adjustments were made for factors that were already known to be important to the development of persistent symptoms and would probably be strongly correlated, such as BMI, smoking, migration background, education level, and comorbidities. Still, with a larger sample size, we might have been able to determine more stable links.

A strength of the present study is the rich sample of participants who prospectively completed multiple questionnaires over a period of 24 months following SARS-CoV-2 infection. The explorative approach used in this study enables us to evaluate the interconnection of different biological, clinical, psychological, and social variables while taking all variables into account. Furthermore, we look at the sensitivity of the results, both through performing sensitivity analysis in which we consider different variables, and through performing bootstrapping on the data to verify that the links found are stable. The hard threshold we set on the stability of the links allows us to focus our attention on the most stable results.

The present study has several limitations that need to be acknowledged. First, putative factors included in the model were measured at different time points during the study period, ranging from 1 to 12 months after initial illness. This was done to prevent questionnaire burden for participants in the acute and subsequent phases of illness. Second, negative life events in the previous 12 months were assessed at month 1, which could result in participants reporting their disease from COVID-19 as a negative life event. Third, fatigue and difficulty concentrating are known symptoms of depression (American Psychiatric Association, 2013) and are part of the instrument used to determine the presence of depressive symptoms (PHQ-9) (Spitzer et al., 1999), making it difficult to untangle associations. Last, the relatively small sample size could have limited our ability to identify stable and causal links.

## Conclusion

From a network perspective, post-COVID-19 sequelae may be sustained by complex interactions among biological, clinical, psychological, and social factors. Using a structural model, we gained a better understanding of the interconnections between these variables. Patients' cognitions can provide an opportunity to improve disease management following SARS-CoV-2 infection.

## Supporting information

Verveen et al. supplementary material Verveen et al. supplementary material

## Data Availability

Data supporting the findings in this manuscript are available from the corresponding author upon reasonable request. The metadata is available at DataVerseNL (Nieuwkerk et al., 2024).

## Supplementary material

The supplementary material for this article can be found at https://doi.org/10.1017/S0033291724002721.
