# Supplementary material for: Long-term sequelae of SARS-CoV-2 two years following infection: exploring the interplay of biological, psychological, and social factors
Source: Psychol Med. 2024 Dec 2;54(15):4408–18. doi: 10.1017/S0033291724002721 (PMC11650182; doi:10.1017/S0033291724002721)
Supplement: Verveen et al. supplementary material [file S0033291724002721sup001.docx]

Supplementary information to: Long-term sequelae of SARS-CoV-2 two years following infection: exploring the interplay of biological, psychological and social factors.

**Tables**

Supplementary Table 1. Baseline characteristics of those who did and did not complete the month 24 visit.

Supplementary Table 2. Stability of edges in Figure 2.

**Figures**

Supplementary Figure 1. Correlation plot of all variables in the structural network model.

Supplementary Figure 2. Structural network model with outcomes assessed at month 12 and inflammatory markers at month 3.

Supplementary Figure 3. Structural network model with outcomes assessed at month 24 and inflammatory markers at month 6.

Supplementary Figure 4. Structural network model with fatigue assessed at month 24 as sole outcome and inflammatory markers at month 3.

# Supplementary Table 1. Baseline characteristics of those who did and did not complete the month 24 visit.

|  | Levels | Not included participants | Included participants | p-value |
| --- | --- | --- | --- | --- |
| Total N (%) |  | N=114 | N=235 |  |
| Sex | Male | 59 (52%) | 137 (58%) | 0.25 |
|  | Female | 55 (48%) | 98 (42%) |  |
| Age, years | Median (IQR) | 48.0 (32.0-64.0) | 52.0 (38.0-62.0) | 0.25 |
| BMI, kg/m2 | Median (IQR) | 27.2 (23.6-31.2) | 25.8 (23.2-29.3) | 0.12 |
| Migration background* | Dutch | 38 (33%) | 156 (66%) | 0.007 |
|  | Non-Dutch, OECD HIC | 12 (11%) | 28 (12%) |  |
|  | Non-Dutch, OECD LMIC | 30 (26%) | 50 (21%) |  |
|  | (Missing) | 34 (30%) | 1 (0%) |  |
| Education level | No formal education | 12 (11%) | 33 (14%) | 0.28 |
|  | Primary education | 23 (20%) | 54 (23%) |  |
|  | Secondary education | 39 (34%) | 147 (63%) |  |
|  | (Missing) | 40 (35%) | 1 (0%) |  |
| Number of COVID-19 high-risk comorbidities** | 0 | 60 (53%) | 129 (55%) | 0.32 |
|  | 1 | 23 (20%) | 58 (25%) |  |
|  | 2 | 17 (15%) | 32 (14%) |  |
|  | 3 | 14 (12%) | 16 (7%) |  |
| Smoking status | Non-smoker | 64 (56%) | 143 (61%) | 0.70 |
|  | Smoker | 6 (5%) | 16 (7%) |  |
|  | Ex-smoker | 27 (24%) | 75 (32%) |  |
|  | (Missing) | 17 (15%) | 1 (0%) |  |
| Hospitalization for COVID-19 | No | 31 (27%) | 133 (57%) | <0.001 |
|  | Yes | 77 (68%) | 102 (43%) |  |
|  | (Missing) | 6 (5%) | 0 (0%) |  |

Abbreviations: BMI, Body Mass Index; COVID-19, coronavirus disease 2019; HIC, high-income country; LMIC, low- and middle-income country; OECD, Organisation for Economic Co-operation, and Development.

* Migration background was based on country of birth of participant and that of their parents and included first and second-generation migrants.

** COVID-related comorbidities are based on World Health Organisation Clinical Management Guidelines (World Health Organization, 2021) and include: cardiovascular disease (including hypertension), chronic pulmonary disease (excluding asthma), renal disease, liver disease, cancer, immunosuppression (excluding HIV, including previous organ transplantation), previous psychiatric illness and dementia.

# Supplementary Table 2. Stability of edges in Figure 2.

| Edge | Stability score (%) |
| --- | --- |
| Fear avoidance and damage avoidance | 100 |
| IL-1β and IL-6 | 100 |
| IL-6 and TNF-α | 100 |
| Fatigue and depressive symptoms | 99 |
| CRP and sCD14 | 98 |
| Resilience and CERQ | 97 |
| Fatigue and physical functioning | 88 |
| IL-2 and IL-1β | 87 |
| Fatigue and concentration | 83 |
| IP-10 and TNF-α | 78 |
| Severity of the initial illness and IP-10 | 74 |
| Concentration and depressive symptoms | 73 |
| Concentration and illness perceptions | 63 |
| Loneliness and depressive symptoms | 62 |
| IL-1β and IL-13 | 61 |
| IL-2 and IL-10 | 60 |
| All-or-nothing behavior and illness perceptions | 59 |
| MCP1 and IP-10 | 58 |
| Loneliness and resilience | 57 |
| All-or-nothing behavior and damage avoidance | 57 |
| IP-10 and IL-10 | 57 |
| Negative life events and IP-10 | 56 |
| Negative life events and resilience | 54 |
| Physical functioning and fear avoidance | 51 |
| MCP1 and sCD163 | 51 |

Abbreviations: CERQ, Cognitive Emotion Regulation Questionnaire; CRP, C-reactive protein; IL, Interleukin; IP-10, Interferon-γ-inducible Protein; MCP-1, Monocyte Chemoattractant Protein; TNF, Tumor Necrosis Factor.

# Supplementary Figure 1. Correlation plot of all variables in the structural network model


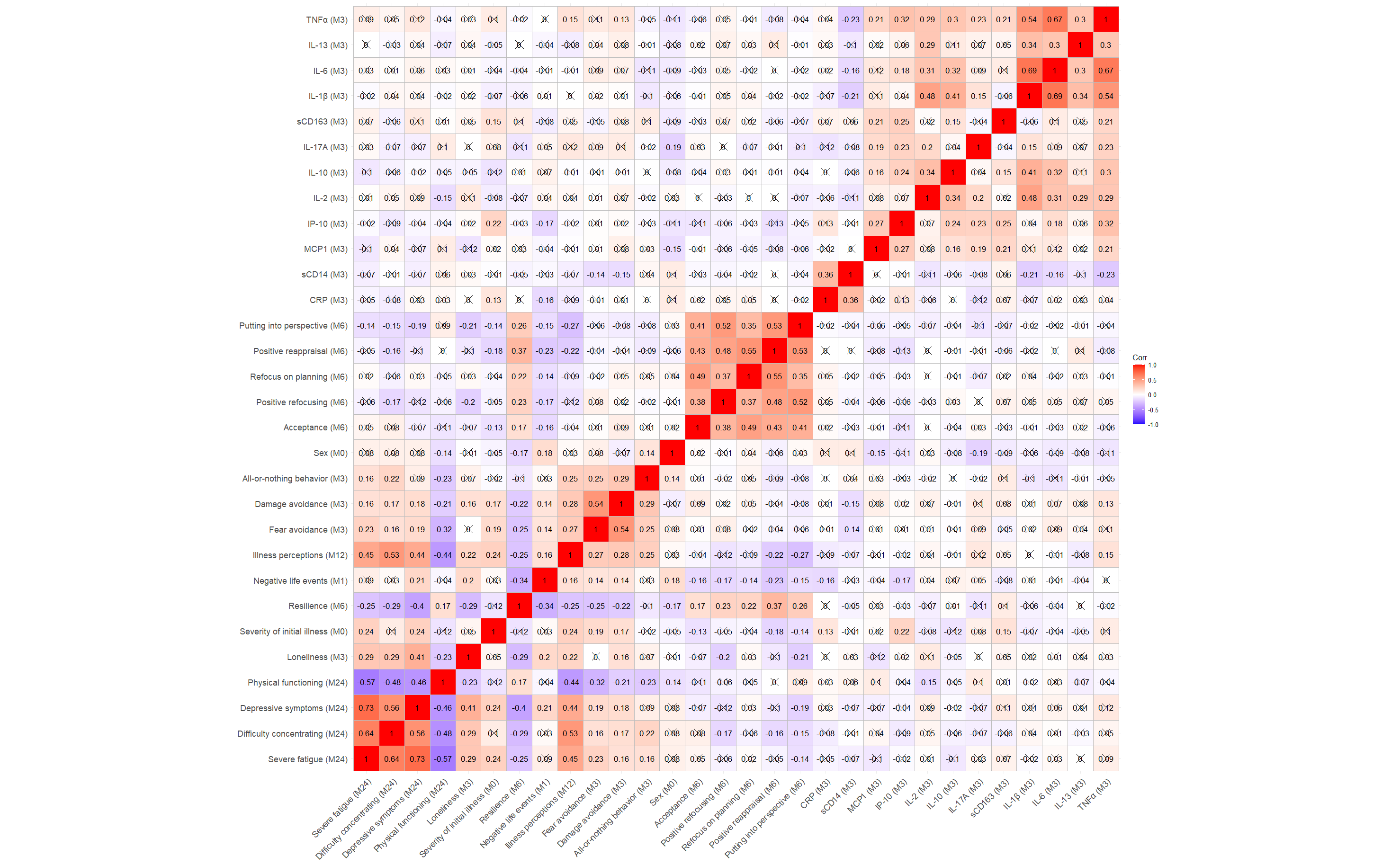


This correlation plot is computed on variables after baseline covariate adjustment (see Table 1 for baseline covariates). Cross signs mean the corresponding correlation coefficient is not significant (p-value > 0.05).

# Supplementary Figure 2. Structural network model with outcomes assessed at month 12 and inflammatory markers at month 3.


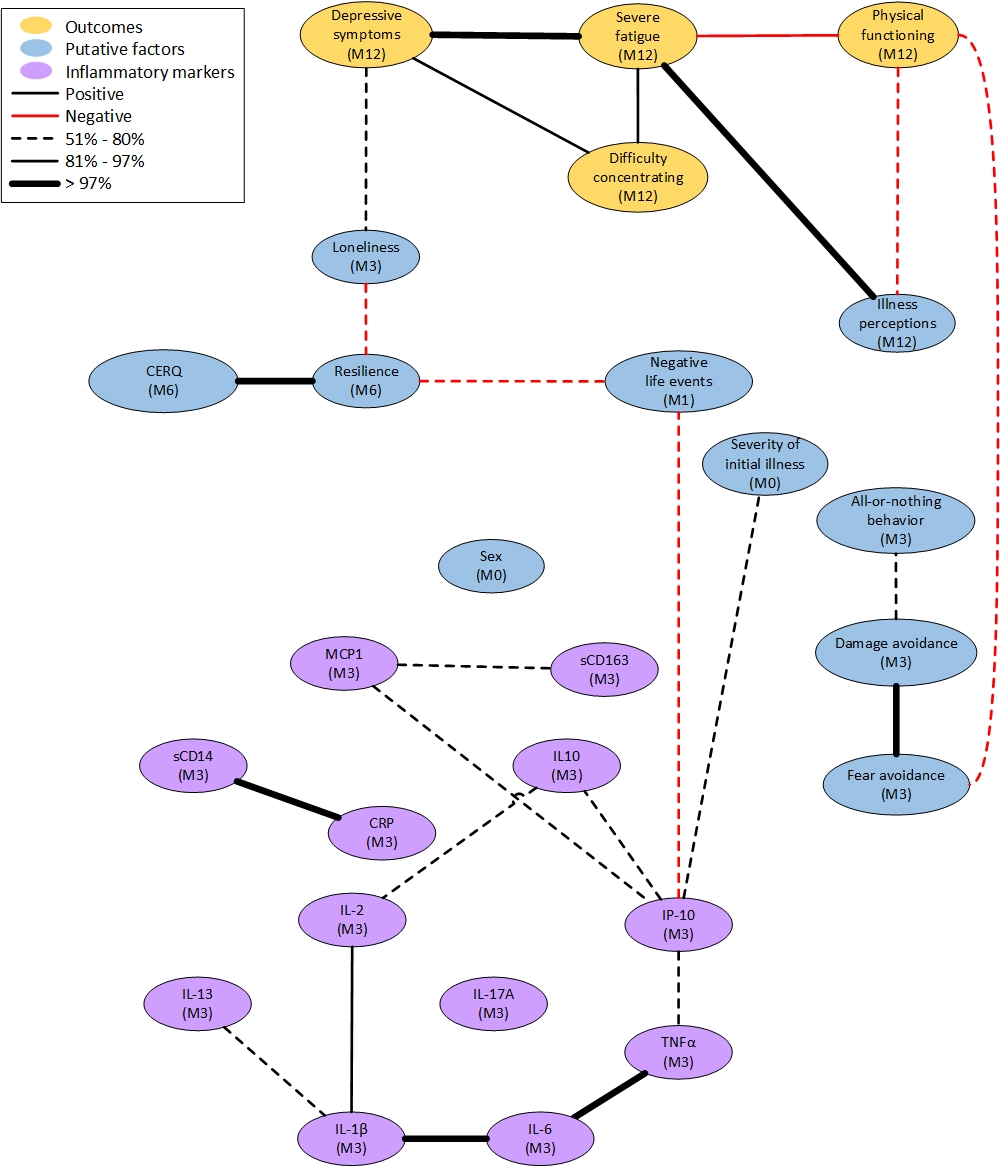


Each line stands for a stable interaction between the two variables it connects, which is not mediated by any other variable in the model. The thickness of a line shows the stability of the interaction: a dashed line has low stability (51%-80%), a solid line is moderately stable (81%-97%), and a bold line very stable (>97%). Red lines refer to a negative correlation between the two connected variables and black lines to a positive correlation. M# gives the month of measurements.

Abbreviations: CRP, C-reactive protein; IL, Interleukin; IP-10, Interferon-γ-inducible Protein; MCP-1, Monocyte Chemoattractant Protein; TNF, Tumor Necrosis Factor.

# Supplementary Figure 3. Structural network model with outcomes assessed at month 24 and inflammatory markers at month 6.


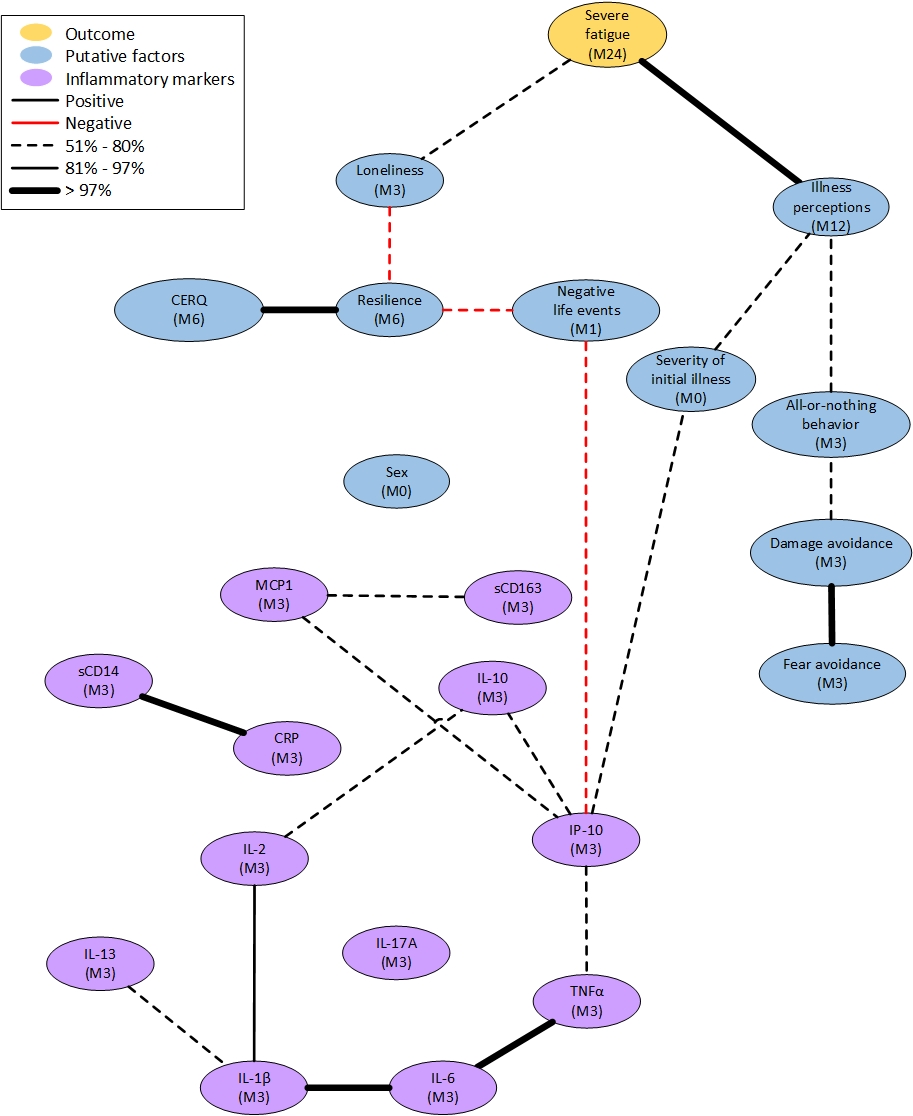

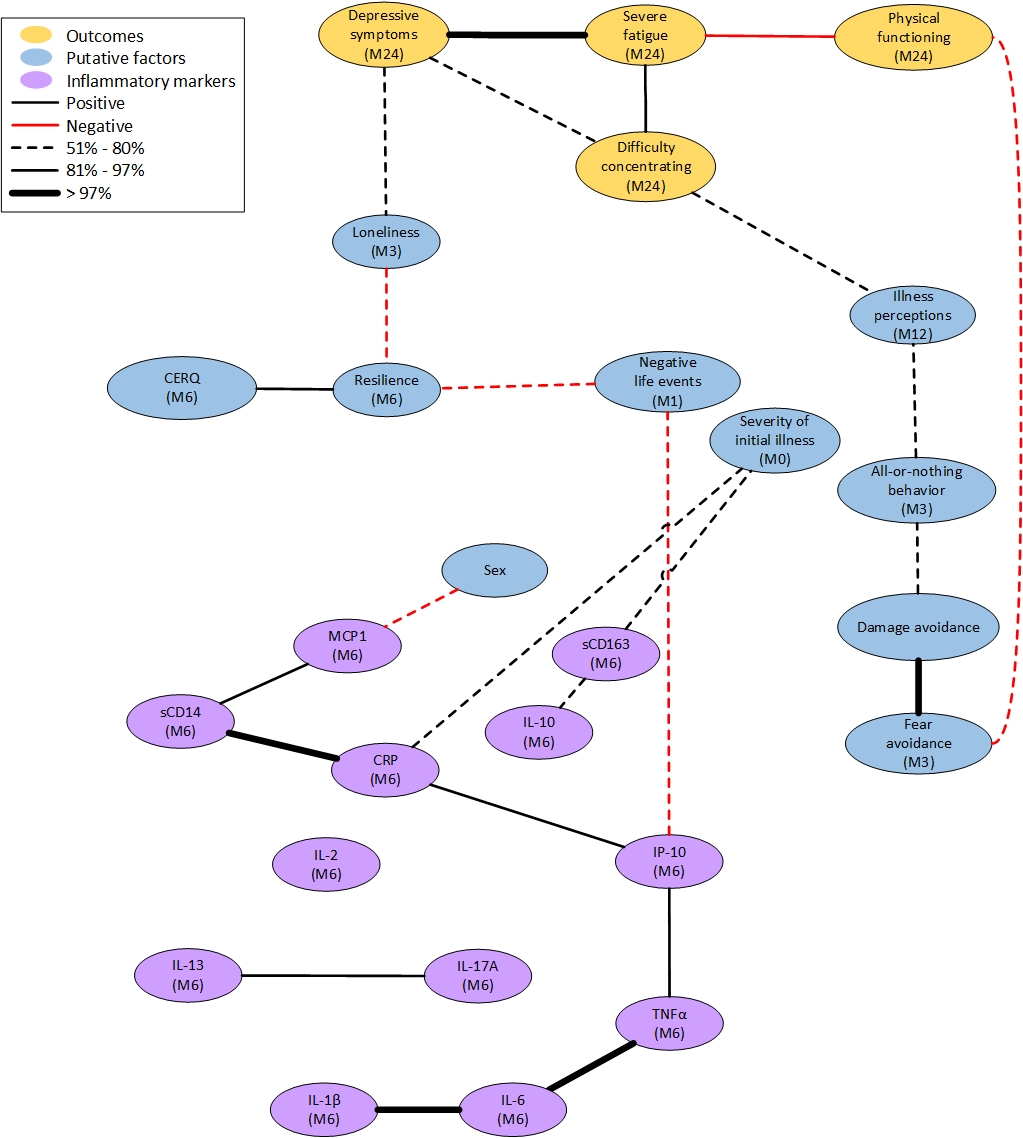


Each line stands for a stable interaction between the two variables it connects, which is not mediated by any other variable in the model. The thickness of a line shows the stability of the interaction: a dashed line has low stability (51%-80%), a solid line is moderately stable (81%-97%), and a bold line very stable (>97%). Red lines refer to a negative correlation between the two connected variables and black lines to a positive correlation. M# gives the month of measurements.

Abbreviations: CRP, C-reactive protein; IL, Interleukin; IP-10, Interferon-γ-inducible Protein; MCP-1, Monocyte Chemoattractant Protein; TNF, Tumor Necrosis Factor.

# Supplementary Figure 4. Structural network model with fatigue assessed at month 24 as sole outcome and inflammatory markers at month 3.


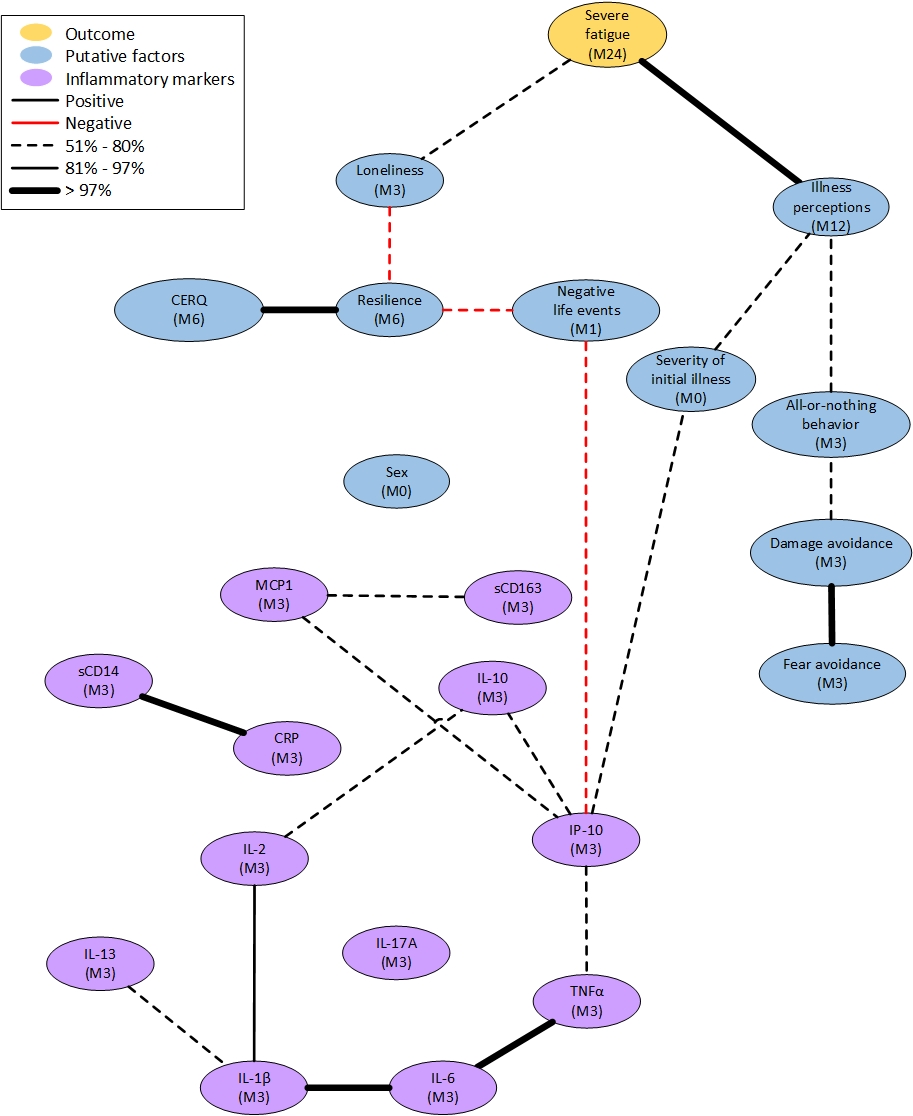


Each line stands for a stable interaction between the two variables it connects, which is not mediated by any other variable in the model. The thickness of a line shows the stability of the interaction: a dashed line has low stability (51%-80%), a solid line is moderately stable (81%-97%), and a bold line very stable (>97%). Red lines refer to a negative correlation between the two connected variables and black lines to a positive correlation. M# gives the month of measurements.

Abbreviations: CRP, C-reactive protein; IL, Interleukin; IP-10, Interferon-γ-inducible Protein; MCP-1, Monocyte Chemoattractant Protein; TNF, Tumor Necrosis Factor.
